# A Generative Adversarial Network Fused with Dual-Attention Mechanism and Its Application in Multitarget Image Fine Segmentation

**DOI:** 10.1155/2021/2464648

**Published:** 2021-12-18

**Authors:** Jian Yin, Zhibo Zhou, Shaohua Xu, Ruiping Yang, Kun Liu

**Affiliations:** ^1^College of Computer Science and Engineering, Shandong University of Science and Technology, Qingdao 266 590, China; ^2^Qingdao Ruisi Intelligent Technology Co., Ltd., Qingdao 266 590, China

## Abstract

Aiming at the problem of insignificant target morphological features, inaccurate detection and unclear boundary of small-target regions, and multitarget boundary overlap in multitarget complex image segmentation, combining the image segmentation mechanism of generative adversarial network with the feature enhancement method of nonlocal attention, a generative adversarial network fused with attention mechanism (AM-GAN) is proposed. The generative network in the model is composed of residual network and nonlocal attention module, which use the feature extraction and multiscale fusion mechanism of residual network, as well as feature enhancement and global information fusion ability of nonlocal spatial-channel dual attention to enhance the target features in the detection area and improve the continuity and clarity of the segmentation boundary. The adversarial network is composed of fully convolutional networks, which penalizes the loss of information in small-target regions by judging the authenticity of prediction and label segmentation and improves the detection ability of the generative adversarial model for small targets and the accuracy of multitarget segmentation. AM-GAN can use the GAN's inherent mechanism that reconstruct and repair high-resolution image, as well as the ability of nonlocal attention global receptive field to strengthen detail features, automatically learn to focus on target structures of different shapes and sizes, highlight salient features useful for specific tasks, reduce the loss of image detail features, improve the accuracy of small-target detection, and optimize the segmentation boundary of multitargets. Taking medical MRI abdominal image segmentation as a verification experiment, multitargets such as liver, left/right kidney, and spleen are selected for segmentation and abnormal tissue detection. In the case of small and unbalanced sample datasets, the class pixels' accuracy reaches 87.37%, the intersection over union is 92.42%, and the average Dice coefficient is 93%. Compared with other methods in the experiment, the segmentation precision and accuracy are greatly improved. It shows that the proposed method has good applicability for solving typical multitarget image segmentation problems such as small-target feature detection, boundary overlap, and offset deformation.

## 1. Introduction

Image multitarget segmentation is one of the hotspots in the field of image processing and artificial intelligence. Essentially, it can be expressed as pixel classification of multitarget with semantic labels, that is, segmenting and describing multitarget of interest in the image by using a set of object categories to classify and mark images at the pixel level [1, 2]. With the continuous development of image analysis theory and deep learning technology, researchers have proposed many effective multitarget image segmentation models and algorithms [3–5]. However, in some complex scenes, the image is affected by noise, offset deformation, gray value distortion, local position effect, and other factors, so the existing methods still have problems such as target omission, position offset, unclear boundary, and so on [6, 7]. At the same time, some image segmentation methods have good results for single-target segmentation, but still not applicable to the complex multitarget image segmentation. Especially, in multi-instance, the accurate detection and segmentation accuracy of small-size targets are difficult to guarantee [8, 9]. There are still challenges in the research of image multitarget detection and segmentation.

At present, image segmentation can be divided into two categories: traditional methods and deep learning methods. Traditional image segmentation algorithms mostly use gray-scale features to segment images [10]. Typical methods include threshold-based method [11], edge-based method [12], and region-based method [13]. However, there are problems such as susceptibility to the contrast of image gray features, poor segmentation of low-resolution and blurred images, and prone to oversegmentation of images. In general, traditional image segmentation methods are greatly affected by subjective factors, and the preprocessing process is complicated. For example, strong prior knowledge is needed to solve the problems such as the selection of seed points during segmentation and the selection of the threshold of the segmentation boundary. At the same time, small errors in the selection of key parameters have a greater impact on segmentation accuracy. These problems make traditional image segmentation algorithms still have many restrictions when applied to image semantic segmentation.

Deep convolutional network is an effective image feature extraction and analysis method, which has been widely used in image classification, image generative, and target detection. Many excellent algorithms have emerged, such as AlexNet [14], ResNet [15], Faster R-CNN [16], and so on. Although deep convolutional networks have powerful feature extraction capabilities, they still have many limitations when applied to image segmentation problems. Take multitarget segmentation of medical images as an example. Medical images intuitively reflect the 2D and 3D morphological characteristics of organs and tissues in specific areas of the human body. The human body has a relatively complex organ and tissue structure, and the anatomical structure of different human bodies is also different among individuals. At the same time, it is easy to be affected by factors such as noise, illumination, and local posture effect, and the image shape and various organ tissue regions are soft boundaries, showing regular or irregular dynamic periodicity with cardiac contraction or relaxation. These factors increase the difficulty of medical image feature differentiation, target detection, and segmentation [17, 18]. Moreover, the pooling layer in the convolutional neural network (CNN) will downsample the input image size and reduce the resolution of the image; the fully connected layer will turn the image features into vectors, destroying the spatial information of the image. Therefore, it still has inadequacy in solving the problem of complex image segmentation. Although these convolution-based methods have achieved certain segmentation results, they ignore the spatial correlation of medical images such as CT and MRI, which is easy to produce nonsmooth and discontinuous segmentation results [19].

In order to solve the limitations of the structure and information processing mechanism of traditional convolutional neural networks in image segmentation problems, Long et al. [20] proposed the fully convolutional network (FCN) model. FCN abandons the fully connected layer of CNN and replaces it with the fully convolution structure. At the same time, it uses the deconvolution operator to restore the image size and introduces a shortcut-connection structure to fuse the high-level characteristics of the network with the low-level features to optimize the segmentation results. At present, most of the deep learning models used in image segmentation are based on the idea of the FCN model. Ronneberger et al. [21] proposed the U-Net model, which retains the convolution and deconvolution structure of the FCN model, but changes the way of fusion of high-level feature map and low-level feature map. The encoding part and the decoding part of U-Net are completely symmetrical, and the connection is realized through channel splicing and then convolution. At present, there are many variants of U-Net models, such as 3D U-Net [22], Res U-Net [23], Dens U-Net [24], and Attention U-Net [25]. In addition, the SegNet model proposed by Badrinarayanan et al. [26] replaces the deconvolution of FCN with an up-pooling method, which makes the upsampling part no longer participate in training while ensuring the segmentation accuracy, reducing the computational complexity. The above methods and mechanisms effectively solve the principle and strategy problems of image segmentation, but in complex image segmentation with noise and content diversity, there are still problems of unstable segmentation effect on low-resolution and fuzzy images and low accuracy of target pixel classification [27].

In the research of low-resolution image high-definition processing, Zhang et al. [28] (2021) build a hierarchical correlation filters model based on the multilevel convolutional features, which can suppress interference of background and similar objects. Chen et al. [29] (2021) proposed an image super-resolution reconstruction method using attention mechanism with the feature map. It uses the information extraction block of feature map attention mechanism to adaptively adjust the channel characteristics, enhance the feature expression ability, and facilitate reconstruction from original low-resolution images to multiscale super-resolution images. For the image inpainting, Chen et al. [30] (2021) proposed a novel image embedding algorithm based on encoder and similarity constraint, which effectively solved the problem of joint context awareness loss in image inpainting and improved the utilization of features. The above works provide good support for fine segmentation of complex images. However, the proposed method will still be affected by obvious blur, and the training time required will also be longer. Then, Chen et al. [31] (2021) proposed image completion algorithm based on the improved total variation minimization method, which can solve the issue mismatching and structure disconnecting in exemplar-based image inpainting.

The generative adversarial network (GAN) proposed by Goodfellow et al. (2014) [32] is a deep learning method based on Nash equilibrium in game theory, including two parts: generator and discriminator. The generator in the GAN model, that is, the generator network model, is mainly used to generate target data. The typical generator structure is a neural network based on deconvolution, which restores the input image size through multiple deconvolution layers' upsampling and finally obtains generated image data. In image segmentation, the generator is essentially a segmentation model. For example, FCN [20], U-Net [21], and SegNet [26] can be selected as the generative network, which receives the input of the original image and takes the predictive segmentation as the output. The discriminator, that is, the adversarial network in GAN, usually uses CNNs as the basic model. Its inputs include the real data and the generator's generated data. The generated data are judged as false, and the real data are judged as true. Through the authenticity judgment, the game learning is performed to optimize the generator's ability to generate data. In mechanism, GAN can reconstruct low-resolution images into super-resolution high-definition images [33] and train the generative model based on the surrounding pixels of the missing part of the image to repair the complete image [34]. If applied to the image segmentation problems in the case of blur, offset, and small target, it can effectively improve the quality and accuracy of image segmentation. Attention mechanism is a target feature enhancement method that is widely studied and applied at present. It can be used as a module of the deep learning model to focus attention on objects of interest [35, 36]. However, most of the existing methods mainly focus on the local pixels of the target area and have low relevance to the image content with a large receptive field [37]. In order to capture the dependence of spatial long-distance information in the image, Wang et al. [38] proposed a nonlocal attention mechanism, the strategy of which is that the characteristic response value at a pixel is equal to the weighted average of the characteristic values at all receptive field points, that is, all points in the larger receptive field are connected to realize global information fusion. The nonlocal attention mechanism connects the understanding of global content with the semantics of local targets, which improves the enlightenment and restriction of target pixel classification. In complex image multiobject segmentation, if the image segmentation mechanism of GAN is combined with nonlocal attention feature enhancement method, it can effectively improve the accuracy of complex image multiobject segmentation and optimize segmentation boundary in mechanism.

Aiming at the poor accuracy of multitarget instance segmentation and small-scale target segmentation in complex images, a generative adversarial network fused with nonlocal attention mechanism is proposed in this paper. The generative network module of the GAN uses the residual network as the basic model for preliminary target segmentation. The nonlocal spatial-channel dual-attention mechanism is added to the output feature map of the residual network to capture the long-distance dependence information of each feature point on the output feature map. The adversarial network module is constructed based on CNNs, which performs masking operations on the original image with prediction segmentation and label segmentation, respectively, and inputs the masking result into the adversarial network. The adversarial network judges the mask result of the predicted segmentation as false, and the mask result of the label segmentation is judged as true. And the generative network judges the mask result of the predicted segmentation as true. Through the game learning between the generative network and the adversarial network, the image segmentation ability of the generative network is optimized. Specifically, firstly, a generative network module is constructed based on residual network and nonlocal attention mechanism for preliminary image segmentation. On this basis, masking operations on the original image with the prediction and label segmentation, respectively, are performed, and the results are input to the adversarial network to optimize the segmentation results. AM-GAN strengthens the extraction and fusion of multiscale features, as well as the distinguishing ability of each instance boundary pixels, to achieve the fine segmentation of multitarget instance regions in complex image and improve the accuracy of small-target segmentation.

Medical images are often blurred due to the influence of the imaging environment and detecting equipment. Meanwhile, the complexity of human organ and tissue structure, the differences of different individual anatomical structures, and the influence of local posture effect also increase the difficulty of medical image feature differentiation and segmentation. In this paper, taking the segmentation of abdominal MRI images as a verification experiment, multitarget tissues such as liver, left/right kidney, and spleen are selected for segmentation and abnormal detection to verify the effectiveness of the model and algorithm.

In this paper, the problems in image segmentation, such as insignificant morphological characteristics of the target image, easy to be affected by noise, gray value distortion and local position effect, and unclear boundary of the target region, are studied. The main motivation is to establish a novel image segmentation model, which can improve the accuracy of complex image fine segmentation in mechanism.

The novelty and main contributions of this paper are as follows:

1. A novel generative adversarial network fused with the attention mechanism (AM-GAN) multitarget image segmentation model is proposed. In mechanism, the image segmentation mechanism of GAN is organically combined with the feature enhancement method of nonlocal attention so that the model and algorithm can automatically learn to focus on the target structure with different shapes and sizes, highlighting the feature usefulness for specific tasks. It can effectively improve the problems of existing image segmentation methods, such as insufficient utilization of correlation information between image voxels, imprecise detection of small-target area, unclear boundary, and overlapping multitarget boundary, and effectively improve the accuracy of complex image multitarget segmentation.
2. The generative module of AM-GAN combines the feature enhancement of nonlocal attention with the feature fusion method of the residual network. In the generative network, the understanding of the global content is linked with the semantics of the local target, and the low-level and high-level feature maps are added through the shortcut-connection structure to achieve feature fusion. In mechanism, it can give play to the guidance and heuristics of target segmentation and refine the segmentation results.
3. In this paper, AM-GAN is proposed as the image multitarget segmentation model. In mechanism, it can use GAN's high-definition processing and repair capabilities to reduce the effects of noise, bias deformation, and gray value distortion. Through the information association and restriction of the nonlocal attention large receptive field to the local target, the continuity of target segmentation is maintained. Meanwhile, the Nash game strategy between generative network and adversarial network is adopted in AM-GAN algorithm to optimize the segmentation results and improve the continuity, smoothness, and accuracy of multitarget segmentation results.

In Section 1, the current challenges and current research status of the complex image multitarget segmentation are reviewed and analyzed. The ideas and algorithm strategies of a novel AM-GAN segmentation model established in this paper are pointed out. In Section 2, the AM-GAN model is established and its theoretical properties are analyzed. In Section 3, the comprehensive learning algorithm of AM-GAN is designed and proposed. In Section 4, multitarget segmentation experiments and result analysis are conducted based on medical images. Finally, the work of the paper is summarized, and the advantages and limitations are pointed out.

## 2. The Generative Adversarial Network Fused with the Dual-Attention Mechanism Segmentation Model

### 2.1. The Generative Adversarial Network Basic Model

**Figure 1:**
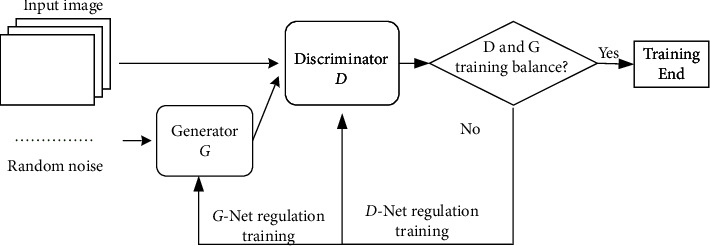
The generative adversarial network.

The generative adversarial network (GAN) basic model in this paper includes two parts: generator and discriminator. The basic structure and information processing flow are shown in Figure 1.

In Figure 1, the generative network module in GAN is mainly used to generate target data. The typical generative network generally is usually the neural network based on deconvolution, such as FCN, U-Net, and SegNet, which recovers to the size of the input image through sampling on multiple deconvolution layers and finally obtains the generated image data. For image segmentation, the generative network is used as an image segmentation model, which receives the input of the original image and takes the predictive segmentation as the output. The adversarial network in GAN usually adopts convolutional neural networks. Its input includes real data and generated data from the generator. Through authenticity judgment and game learning with the generator, the ability of the generator to generate data is optimized.

The gray value of the input image to generative network is recorded as the random variable *x*, the distribution it obeys is set to *P*(*x*), and the noise data *z* obeys the uniform distribution. The generator maps the noise data *z* to *G*(*z*), and the authenticity discrimination probability of the image random variable *x* is *D*(*x*).

In learning, GAN is optimized according to the principle that the generative model maximizes log  *D*(*x*) and the discrimination model minimizes log(1 − *D*(*G*(*z*))). The objective function is defined as follows:

(1) $$\begin{array}{c} \underset{G}{\min}\underset{D}{\max}V\left(D,G\right)=E_{x∼P\left(x\right)}\left[\log\;D\left(X\right)\right]+E_{z∼P\left(x\right)}\left[\log\left(1-D\left(G\left(Z\right)\right)\right)\right]. \end{array}$$

### 2.2. Nonlocal Attention Mechanism

The core idea of nonlocal attention mechanism is that the characteristic response value at a pixel is equal to the weighted average of the characteristic values at all points, that is, all points in the receptive field are connected, and the dependency relationship between pixels based on spatial distance is established to realize global information fusion. The calculation formula [38] is

(2) $$\begin{array}{c} y_{i}=\frac{1}{C\left(x\right)}\sum_{\forall j}f\left(x_{i},x_{j}\right)g\left(x_{j}\right), \end{array}$$

where *i* represents the output position index, *j* represents any position of the input, *x* represents the input, *y* represents the output with the same size as *x*, *f*(·) is the similarity measurement function between pixels, *g*(·) represents the feature mapping of *j*, and *C*(*x*) is the normalization factor. (*f*) and *g* can be implemented in many ways. If *g* is a Gaussian function, its expression is

(3) $$\begin{array}{c} f\left(x_{i},x_{j}\right)=e^{x_{i}^{T}x_{j}}, \end{array}$$

(4) $$\begin{array}{c} C\left(x\right)=\sum_{\forall j}f\left(x_{i},x_{j}\right). \end{array}$$

The operation in equation (3) represents the difference amplified exponentially after multiplying the two vector matrices, and equation (4) is the mathematical expression of the normalization function *C*(*x*).

Consider an extended form of equation (3). Firstly, the vector *x* is embedded into spatial mapping, that is, the two vectors are mapped to different feature spaces, and then, the Gaussian function is used to measure the similarity. The specific form is

(5) $$\begin{array}{c} f\left(x_{i},x_{j}\right)=e^{\theta {\left(x_{i}\right)}^{T}\varphi \left(x_{j}\right)}. \end{array}$$

The vector dot product of function *f* can be expressed as

(6) $$\begin{array}{c} f\left(x_{i},x_{j}\right)=\theta {\left(x_{i}\right)}^{T}\varphi \left(x_{j}\right). \end{array}$$

Now, the normalization function *C*(*x*) is equal to *N*, and *N* is the number of positions of *x*.

Based on the paired function form in the relational network proposed by Santoro et al. [39], the function *f* can be expressed as a cascaded form:

(7) $$\begin{array}{c} f\left(x_{i},x_{j}\right)=Relu\left(w_{f}^{T}\left[\theta \left(x_{i}\right),\varphi \left(x_{j}\right)\right]\right), \end{array}$$

where [,] represents cascade and *w*_*f*_^*T*^ represents the weight vector that projects the cascade vector to the scalar.

The nonlocal attention mechanism is integrated into convolutional neural network to form a general nonlocal block. The module can be integrated into any neural network structure. The definition of nonlocal block is as follows:

(8) $$\begin{array}{c} z=w_{z}y+x, \end{array}$$

where *z* is the output of the attention module, *y* is the output of equation (2), *w*_*z*_ is the weight matrix of the number of restored input channels, and *x* is the input.

**Figure 2:**
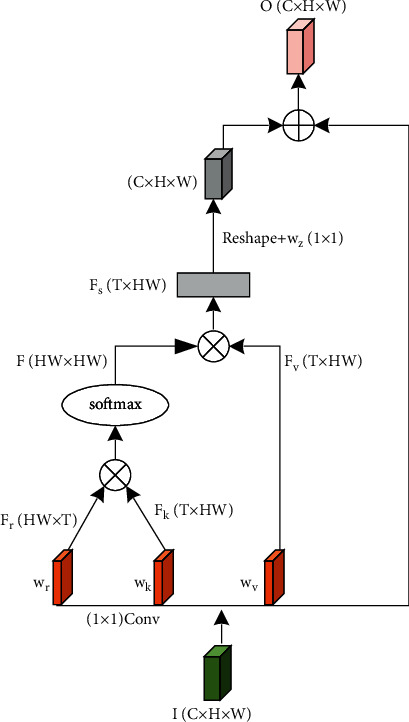
The structure of nonlocal block.

The structure of nonlocal block is shown in Figure 2.

In Figure 2, the nonlocal block first performs feature mapping on the input matrix *I*(*C* × *H* × *W*) with a 1 × 1 convolution, that is, the mapping operation of the functions *g*, *θ*, and *φ* in (3) and (5), to get matrices *w*_*r*_, *w*_*k*_, and *w*_*v*_. Then, the matrix *w*_*k*_ is deformed and transposed into a matrix *F*_*k*_(*HW* × *T*), and *w*_*r*_ is transformed into a matrix *F*_*r*_(*T* × *HW*). *F*_*k*_ is multiplied by *F*_*r*_ and then normalized by Softmax to obtain the similarity measure matrix *F*(*HW* × *HW*). Then, multiply the deformed matrix *F*_*v*_(*T* × *HW*) from *w*_*v*_ and the matrix *F* to obtain the characteristic response matrix *F*_*s*_(*T* × *HW*). Finally, *F*_*s*_ is deformed and multiplied by the convolution kernel *w*_*z*_ to restore the original number of channels. The operation result is added to the input matrix *I*(*C* × *H* × *W*) to obtain the output *O*(*C* × *H* × *W*), and the final output feature map is a feature map with enhanced global information dependence.

### 2.3. Residual Generation Network Based on Dual-Attention Mechanism

**Figure 3:**
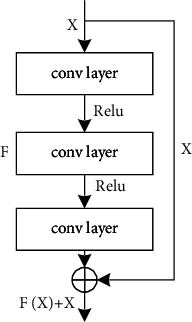
Residual structure.

In the complex images' multitarget segmentation, if the number of image instance targets is large and the difference of individual gray value is small, the neural network needs to have strong ability of feature extraction, fusion, and recognition. In this paper, the residual network is used as the main body of the image segmentation model, and the shortcut-connection structure is added between different convolution layers, that is, the input of the upper network is directly superimposed with the output of the lower network at the element level, so as to reduce the loss of feature information and realize feature fusion of different levels. In this way, the network can still maintain good convergence properties in the deep case. The residual structure is shown in Figure 3.

**Figure 4:**
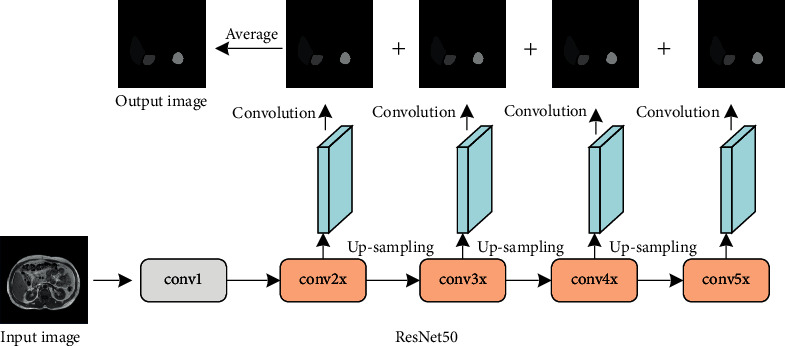
The segmentation model based on residual network.

At present, there are many classical residual network models, such as ResNet18, ResNet34, and ResNet50 [16]. In practice, the selection of the model is mainly based on the requirements of input image size, quality, and segmentation accuracy. Generally, the deeper the network, the stronger the feature extraction ability. In this paper, ResNet50 is selected as the basic model according to the problem of small-target region detection in complex image multitarget segmentation. The structure of segmentation model based on ResNet50 is shown in Figure 4.

In Figure 4, the segmentation model is divided into six parts. (1) 7 × 7 convolution with step size of 2 and the number of output image channels is 64. (2) The pooling operation with step size of 2 and the 3 × 3 sliding window cascades three residual blocks. Each residual block contains three convolution layers. The first and third are 1 × 1 convolution to adjust the number of channels of the feature maps. The second is 3 × 3 convolution with a step size of 1. Equations (3) to (5) are stacked residual blocks. The last one includes average pooling with a step size of 1 and 7 × 7 sliding window, as well as fully connection and Softmax classification. Extract the output from the second to the fifth part of the model, and then, upsample the four output feature maps for prediction segmentation.

**Figure 5:**
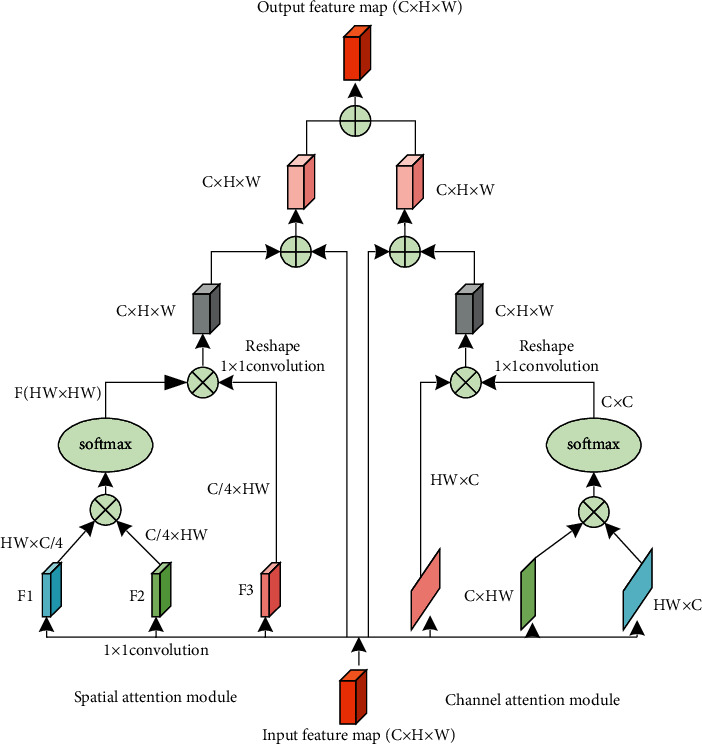
Dual-attention mechanism.

In practice, only relying on the residual network to perform image multitarget segmentation will result in the blurring of the segmentation boundary and the loss of small-target information, that is, the residual network cannot make full use of the image feature information to segment the image. In this paper, based on the residual network, a nonlocal attention mechanism is introduced to construct a dual-attention mechanism model that can integrate spatial and channel attention. The structure is shown in Figure 5.

In Figure 5, the spatial-attention module is used on a nonlocal mechanism to strengthen the dependency between all pixels on the feature maps, which is represented by a similar weight matrix. In this module, the output feature map of the residual network first undergoes 1 × 1 convolution to obtain three feature maps *F*1, *F*2, and *F*3, which have the same width and height as the input image, but the number of channels is reduced to 1/4 of the original. On this basis, *F*1 is transposed and multiplied by *F*2 and then processed by Softmax normalization to obtain the interpixel similarity matrix *F*(*HW* × *HW*). *F* is multiplied by *F*3, and the original input is added to obtain the feature map after spatial feature optimization.

The channel-attention mechanism is used to capture the interdependence between any two channels and update the value in one channel by using the weighted average of all channels. Its implementation steps are similar to the spatial-attention module. The feature maps obtained by the spatial-attention module and the channel-attention module are added and fused to generate a segmented image strengthened by the attention mechanism.

In Figure 5, the mathematical expression of Softmax is

(9) $$\begin{array}{c} \text{softmax}\left(z_{i}^{l}\right)=\frac{e^{z_{i}^{l}}}{\sum_{p=0}^{\left(H\times W\right)-1}e^{z_{p}}}, \end{array}$$

where *z*_*i*_^*l*^ represents the *i*^*th*^ pixel value in the *l*^*th*^ column of the feature map *F*. This formula normalizes the similarity measure matrix *F* by column so that the weight value of each column is within the interval [0,1].

**Figure 6:**
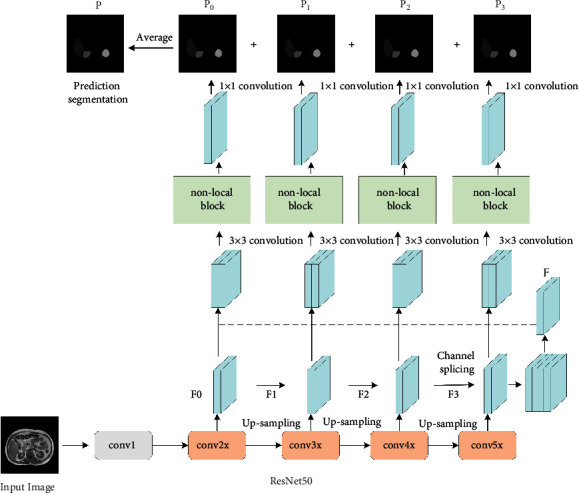
The generate network model.

The nonlocal attention mechanism is combined with the residual network model to construct the generator module in the GAN. The structure is shown in Figure 6.

In Figure 6, the generator module receives image data input, and the input image is extracted features by the ResNet50 network based on the dual-attention mechanism; four output feature maps at different levels are obtained, denoted as *F*0, *F*1, *F*2, and *F*3. The *F*1, *F*2, and *F*3 feature maps are upsampled to the same size as *F*0, and then, the four output feature map channels are spliced and 3 × 3 convolution is performed to obtain the fused feature map *F*. The fusion feature map *F* is spliced with *F*0, *F*1, *F*2, and *F*3 at channel level, respectively, and then, input into the self-attention mechanism module after 3 × 3 convolution to obtain four prediction segmentation maps. They are added and fused and averaged, and finally, the image multitarget prediction segmentation map is obtained.

### 2.4. The Adversarial Network Model Based on Convolutional Network

**Figure 7:**
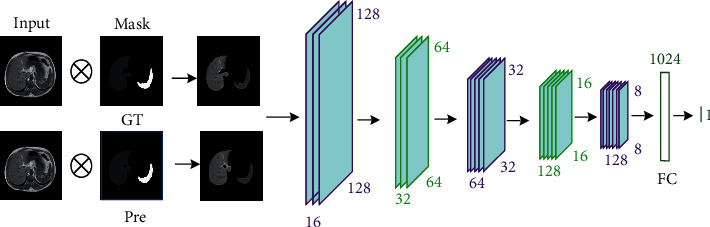
The adversarial network model.

In GAN, the adversarial network module penalizes the lost details and small-size target information of the network through game learning, which makes the multitarget images segmented by the generative network more accurate. In this paper, the adversarial network module in GAN is constructed based on CNN, and its structure is shown in Figure 7.

In Figure 7, the adversarial network model includes two inputs: one is dot-product image of segmentation label and the original image, and the other is dot-product image of generative network and the original image. Dot-product operation is a mask operation on the original image to obtain the area of label and prediction segmentation on the original image. The adversarial network performs feature extraction on the obtained detection area, distinguishes whether the input is a real or predicted segmentation area, and optimizes the segmentation result through the adversarial learning with the generative network. In this paper, the CNN in the adversarial network model contains a total of 5 convolutional layers, 5 pooling layers, and two fully connected layers. The size of the convolution kernel is 3 × 3, and the step size is 1 × 1, that is, the convolution operation does not change the size of the input. The pooling layer is the maximum pooling with a size of 2 × 2, and the step size is 2 × 2, which reduces the input resolution by half. The ReLu function is selected as the activation function, and the Sigmoid function is used for classification operations.

### 2.5. The Generative Adversarial Network-Fused Attention Mechanism

**Figure 8:**
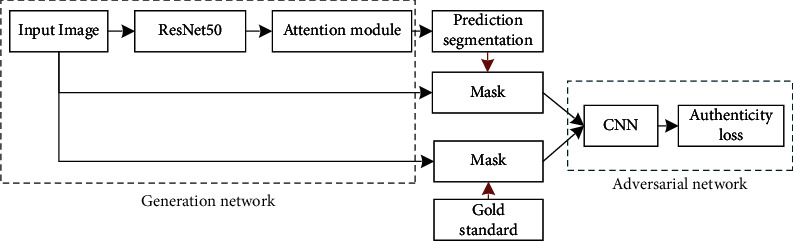
The overall structure of the generative adversarial network segmentation model.

In this paper, a generative adversarial network segmentation model fused with attention mechanism (AM-GAN) is proposed and the overall structure is shown in Figure 8.

In Figure 8, the gold standard is an accurate result of manual segmentation by experts. In AM-GAN, the generative network is composed of the ResNet50 model and the nonlocal dual-attention mechanism module. It takes the original image as input and the predicted segmentation as output. The adversarial network is composed of CNNs. Its inputs include the mask of the predicted segmentation and the original image and the mask of the gold standard and the original image. The mask of the predicted segmentation is judged as false (marked as 0), and the mask of the gold standard is judged to be true (marked as 1).

**Figure 9:**
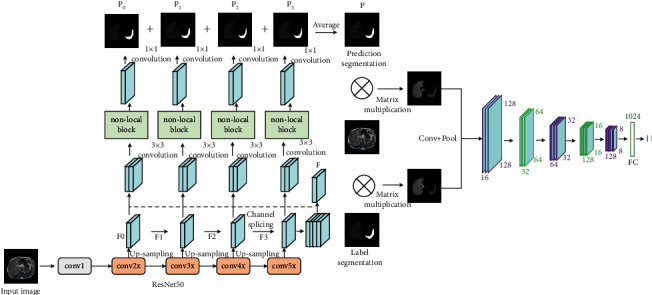
The generative adversarial network segmentation model fused with attention mechanism.

Combining the generative network based on the residual network and the nonlocal dual-attention mechanism with the adversarial network based on the CNNs to build a generative adversarial network model for the multitarget image segmentation. After AM-GAN is trained to reach the optimum, the model used for image segmentation is a generator network. The structure of the AM-GAN segmentation model is shown in Figure 9.

As shown in Figure 9, in the generator network, the *conv*2*x*, *conv*3*x*, *conv*4*x*, and *conv*5*x* parts of the ResNet50 based on the dual-attention mechanism all have a prediction segmentation output, and the final prediction segmentation of the generator network is the average value of the prediction segmentation of these four parts. The input of the generator is subjected to feature extraction through the extended ResNet50 network, and the rough feature map is obtained by upsampling. The calculation formula of the upsampling output *F*_1_ of the *conv*3*x* part in the extended ResNet50 network is

(10) $$\begin{array}{c} F_{1}=\text{Upsample}\left(\text{Relu}\left(BN\left(w_{1}\left(conv3x\left(I\right)+b_{1}\right)\right)\right)\right), \end{array}$$

where *I* represents the input image, conv3*x*(*I*) represents the convolution output of the *conv*3*x* part, *w*_1_ represents the 1 × 1 convolution, whose purpose is to reduce the number of channels of the convolution output, *b*_1_ represents the bias, *BN*(·) represents the batch normalization, *Relu*(·) is the activation function, and Umsample(·) is the interpolation upsampling. In this paper, the bilinear interpolation algorithm is used to upsample the feature map, and the output is *F*_1_. Using the same algorithm, the upsampled output *F*_2_ and *F*_3_ of the *conv*4*x* and *conv*5*x* parts can be obtained. The output *F*_0_ of the *conv*2*x* part does not need to be upsampled, and the sizes of *F*_1_, *F*_2_, and *F*_3_ are the same as *F*_0_. After feature extraction and upsampling of the input image, the output information of each part is fused. The calculation formula of the fusion feature map *F* is

(11) $$\begin{array}{c} F=\text{Relu}\left(w_{3}\left(\text{Relu}\left(w_{2}\left(\text{cat}\left(F_{0},F_{1},F_{2},F_{3}\right)\right)+b_{2}\right)\right)+b_{3}\right), \end{array}$$

where cat(·) represents the splicing operation of feature map channel, *w*_2_ is a 3 × 3 convolution, which is used to fuse the information between the four feature maps, *w*_3_ is a 1 × 1 convolution, which is used to reduce the number of channels of the fusion feature map, *b*_2_ and *b*_3_ are biases, and Relu(·) is the activation function. After the information of each part is fused, the feature map is input into the dual-attention mechanism module for feature enhancement. The calculation formula of the predicted output *P*_1_ of the *conv*3*x* module is

(12) $$\begin{array}{c} P_{1}=\text{Upsample}\left(w_{4}\left(p\text{Attention}\left(\text{cat}\left(F,F_{1}\right)\right)+c\text{Attention}\left(\text{cat}\left(F,F_{1}\right)\right)\right)+b_{3}\right), \end{array}$$

where *p*Attention(·) represents the spatial-attention mechanism module, *c*Attention(·) represents the channel-attention mechanism module, *w*_4_ represents 1 × 1 convolution, which is used to convert the number of channels into the number of classification categories, Upsample(·) represents a bilinear interpolation up-sampling operation, which is used to restore the size of output feature map to the size of the input image to obtain a predicted segmented image. Using the same method, the predicted output *P*_0_, *P*_1_, and *P*_3_ can be obtained. Finally, *P*_0_, *P*_1_, *P*_2_, and *P*_3_ are added and averaged to obtain the predicted segmented image. The calculation formula is

(13) $$\begin{array}{c} P=\text{avg}\left(P_{0}+P_{1}+P_{2}+P_{3}\right). \end{array}$$

Equation (13) is the calculation expression for predicting segmentation *P*, where avg(·) represents the average operation.

In the GAN segmentation model established in this paper, the data processing capability of the ResNet50 extended based on the nonlocal dual-attention mechanism can mechanically ensure that the image multitarget features can be accurately extracted, while nonlocal attention mechanism also strengthens the output feature map of the ResNet50, which can further improve the accuracy of segmentation. The authenticity judgment of the adversarial network can guide the segmentation network to avoid the loss of detailed information as much as possible and optimize boundary segmentation and small-target segmentation.

## 3. The Learning Algorithm

### 3.1. The Loss Function

In the complex images' multitarget segmentation, it is necessary to calculate classification errors of multiclass pixel. In this paper, the multiclassification cross-entropy loss [40] is used as the loss function of the generative network, which is defined as follows:

(14) $$\begin{array}{c} L_{\text{mec}}\left(x,y\right)=-\sum_{i=1}^{\left(H\times W\right)}\sum_{c=1}^{C}x_{ic}\text{ln}y_{ic}. \end{array}$$

Equation (14) represents the multiclassification cross-entropy loss of the label image *x* and the predicted segmentation *y*, where (*H*, *W*, *C*) represents the length, width, and number of channels of the image.

In the AM-GAN segmentation model, the adversarial network uses the authenticity loss to perform game learning with the generative network, which is essentially a binary classification problem. Therefore, the two-classification cross-entropy loss [41] is used as the loss function of the adversarial network, which is defined as follows:

(15) $$\begin{array}{c} L_{\text{bec}}\left(x,y\right)=-\left[x\;\ln\;y+\left(1-x\right)\ln\left(1-y\right)\right]. \end{array}$$

Equation (15) represents the two-classification cross-entropy loss, where *x* represents the classification label and *y* represents the classification probability output of the adversarial network.

The loss function of the generative network is defined as follows:

(16) $$\begin{array}{c} G\left(x_{n},y_{n},\theta_{g}\right)=L_{\text{mec}}\left(g\left(x_{n},\theta_{g}\right),y_{n}\right)+\lambda L_{\text{bec}}\left(d\left(g\left(x_{n},\theta_{g}\right),x_{n}\right),1\right), \end{array}$$

where *x*_*n*_ is the input image, *y*_*n*_ is the label segmentation, and *θ*_*g*_ is the training parameter set of the generative network. According to equation (16), the loss of generative network is composed of two parts, that is, the multiclassification cross-entropy loss of prediction segmentation and label segmentation and the loss of adversarial network which predict segmentation. The hyperparameter *λ* is used to balance these two losses. When optimizing the generative network, we minimize the objective function.

The loss function of the adversarial network is defined as

(17) $$\begin{array}{c} D\left(x_{n},y_{n},\theta_{d}\right)=L_{\text{bec}}\left(d\left(x_{n},y_{n},\theta_{d}\right),1\right)+L_{\text{bec}}\left(d\left(x_{n},g\left(x_{n}\right),\theta_{d}\right),0\right), \end{array}$$

where *θ*  _*d*_ represents the training parameter set of the adversarial network. From (17), the loss function of the adversarial network consists of two parts. The first is the two-classification cross-entropy loss of the mask map of input image *x*_*n*_ and the label segmentation *y*_*n*_, which is judged to be true (that is, the value is 1). The second is the two-classification cross-entropy loss of the mask map of input image *x*_*n*_ and the label segmentation *g*(*x*_*n*_), which is judged to be false (that is, the value is 0). When optimizing the adversarial network, we minimize the loss function.

The loss function of GAN is composed of the loss of the generative network and the loss of the adversarial network. The calculation expression is

(18) $$\begin{array}{c} V\left(G,D\right)=G\left(x_{n},y_{n},\theta_{g}\right)+D\left(x_{n},y_{n},\theta_{d}\right). \end{array}$$

### 3.2. The Training Process

AM-GAN adopts the method of alternate training of generative network and adversarial network. In order to ensure the stability of training, the training times of the discriminating module are generally more than that of the generative module. In this paper, the minibatch gradient descent (MBGD) algorithm is used for AM-GAN training. The specific process is as follows:

(1) Randomly sample *n* samples *z*_*n*_ from noise samples, and *n* samples *x*_*n*_ from real samples.
(2) Gradient ascent algorithm is used to update the adversarial network:
  (19) $$\begin{array}{c} \nabla_{\theta_{d}}\frac{1}{n}\sum_{i=1}^{n}\left(\log\left(D\left(x^{i}\right)\right)+\log\left(1-D\left(G\left(z^{i}\right)\right)\right)\right). \end{array}$$
(3) Repeat steps (1) and (2) *k* times to update the adversarial network *k* times.
(4) Sampling *n* generated samples from the noise samples, and we update the generative network once using gradient descent algorithm:
  (20) $$\begin{array}{c} \nabla_{\theta_{s}}\frac{1}{n}\sum_{i=1}^{n}\log\left(1-D\left(G\left(z^{i}\right.\right)\right). \end{array}$$
(5) Sequentially, we repeat the above steps until the model training is stable and optimal.

The specific algorithm implementation is as follows:

Step 1: determine and initialize the GAN training parameter set. The training parameter set of the generated network is *θ*_*g*_=(*w*_0_, *w*_1_,…, *w*_*m*_, *b*_0_, *b*_1_,…, *b*_*m*_, *g*, *θ*, *φ*, *w*_*z*_, *w*_*c*_), where (*w*_0_, *w*_1_,…, *w*_*m*_) is the set of convolution kernel weight of extended ResNet50 network, (*b*_0_, *b*_1_,…, *b*_*m*_) is the set of biases, *g*, *θ*, *φ*, and *w*_*z*_ are 1 × 1 convolution kernel weights of spatial-attention mechanism, and *w*_*c*_ is 1 × 1 convolution kernel weights of channel-attention mechanism. The training parameter set of the adversarial network is *θ*  _*d*_=(*w*_0_, *w*_1_,…, *w*_*n*_, *b*_0_, *b*_1_,…, *b*_*n*_), where (*w*_0_, *w*_1_,…, *w*_*n*_) is the set of convolution kernel weights of CNN, and (*b*_0_, *b*_1_,…, *b*_*n*_) is the set of biases.
Step 2: randomly select *N* image samples *x*_*n*_ from the sample set, and select corresponding *N* label segmentation samples *y*_*n*_.
Step 3: calculate the loss of the adversarial network: ∇*L*(*θ*  _*d*_)=1/*N*∑_*n*=1_^*N*^[*L*_*bec*_(*d*(*x*_*n*_, *y*_*n*_, *θ*  _*d*_), 1)+*L*_*bec*_(*d*((*x*_*n*_, *g*(*x*_*n*_), *θ*  _*d*_)), 0)].
Step 4: calculate the training parameter gradient according to the chain rule, *a*_*i*_=*σ*(*z*_*i*_)=*σ*(*w*_*i*_*a*_*i*−1_+*b*_*i*_). *σ* is the activation function, *a*_*i*_ is the input feature map of the *i*^*th*^ layer, *z*_*i*_ is the feature map of the *i*^*th*^ layer after convolution, *w*_*i*_ is the weight of the *i*^*th*^ layer, and *b*_*i*_ is the bias of the *i*^*th*^ layer.
The formula for calculating the weight gradient using the chain rule is
  (21) $$\begin{array}{c} \frac{\partial L\left(\theta_{d}\right)}{\partial w_{i}}=\frac{\partial L\left(\theta_{d}\right)}{\partial z_{i}}\cdot a_{i-1}=\sigma^{\prime }\left(z_{i}\right)\cdot {\left(w_{i+1}\right)}^{T}\cdot \left(\frac{\partial L\left(\theta_{d}\right)}{\partial z_{i+1}}\right)\cdot a_{i-1}, \end{array}$$
where *σ*′(·) is the derivative of the activation function and (·)^*T*^ represents the matrix transpose.
The formula for calculating the bias gradient using the chain rule is
  (22) $$\begin{array}{c} \frac{\partial L\left(\theta_{d}\right)}{\partial b_{i}}=\frac{\partial L\left(\theta_{d}\right)}{\partial z_{i}}=\sigma \left(z_{i}\right)\cdot {\left(w_{i+1}\right)}^{T}\cdot \left(\frac{\partial L\left(\theta_{d}\right)}{\partial z_{i+1}}\right). \end{array}$$
Step 5: use the MBGD optimizer to update the convolution weights and biases of the adversarial network:
  (23) $$\begin{array}{c} w_{i}^{t}=w_{i}^{t-1}-\eta \frac{1}{N}\sum_{n=1}^{N}\frac{\partial L\left(\theta_{d}^{t-1}\right)}{\partial w_{i}^{t-1}}, \end{array}$$
  (24) $$\begin{array}{c} b_{i}^{t}=b_{i}^{t-1}-\eta \frac{1}{N}\sum_{n=1}^{N}\frac{\partial L\left(\theta_{d}^{t-1}\right)}{\partial b_{i}^{t-1}}. \end{array}$$
Step 6: repeat Step 2 Step 5 *k* times.
Step 7: randomly select *N* image samples *x*_*n*_, and select corresponding *N* label segmentation samples *y*_*n*_.
Step 8: calculate the loss ∇*L*(*θ*_*g*_) of the generative network:
  (25) $$\begin{array}{c} \nabla L\left(\theta_{g}\right)=\frac{1}{N}\sum_{n=1}^{N}\left[L_{\text{mec}}\left(g\left(x_{n},\theta_{g}\right),y_{n}\right)\right.\\ \left.+\lambda L_{\text{bec}}\left(d\left(g\left(x_{n},\theta_{g}\right),x_{n}\right),1\right)\right]. \end{array}$$
Step 9: use the MBGD optimizer to update the parameters *θ*_*g*_ of the generation network.
Step 10: if the network converges to the optimal, then the training ends; otherwise, it returns to Step 2.

The epoch of training is set to 200 and the batch size is 10. According to the above algorithm process, the pseudocode of the training algorithm is shown in Algorithm 1.

## 4. The Experiment and Result Analysis

### 4.1. The Experiment Dataset

**Figure 10:**
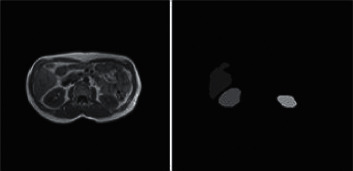
Data sample from CHAOS.

The dataset comes from the Combined Healthy Abdominal Organ Segmentation (CHAOS) competition dataset [42]. The CHAOS dataset contains the abdomen MRI images. In the experiment, the abdominal MRI abdominal images containing the label information of the liver, left/right kidney, and spleen were selected as the sample set. The typical abdomen image and label segmentation image are shown in Figure 10.

The experimental dataset contains 38 groups of abdominal MRI images, and each group has 26 slices with a size of 256 × 256, totaling 988 slices. The dataset is divided into a training set, a validation set, and a test set. The training set includes 30 groups of slices, the validation set includes 2 groups of slices, and the test set includes 6 groups of slices. Due to the small number of samples in the dataset, random rotation, mirroring, and other operations are used to enhance the data in the experiment. The final number of slices in the training set is expanded to 3120, the verification set is expanded to 104, and the test set is expanded to 312. The sample distribution of dataset is shown in Table 1.

### 4.2. The Model Structure and Parameter Settings

The training strategy of the AM-GAN is to alternate training between the generative network and the adversarial network. In order to ensure the stability of training, the ratio of the training times of the generative network and the adversarial network is set to 1 : 6, that is, the adversarial network training is performed 6 times first, and then, the generative network is trained once.

The size of the convolution kernel of the extended ResNet50 in the generative network is all 3 × 3, the step size of the downsampling is 2 × 2, and the other step size is 1 × 1. The activation function adopts the ReLu function, and the balance coefficient *λ* of the loss function of generative network is set to 0.2. The adversarial network includes 5 convolutional layers, 5 pooling layers, and 2 fully connected layers. The convolution kernel size of the convolutional layer is set to 3 × 3, and the step size is 1 × 1. The pooling window size of the pooling layer is set to 2 × 2, and the step size is 2 × 2. The Sigmoid function is used as the classification function. The learning rate of the MBGD optimizer is set to 0.01, and the weight parameters are initialized with truncated normal distribution. The number of training iterations is 200, and the batch of one training is 10. The experimental environment is Linux system with NVIDIA GeForce RTX 2080Ti GPU.

### 4.3. The Experiment Result and Analysis

**Figure 11:**
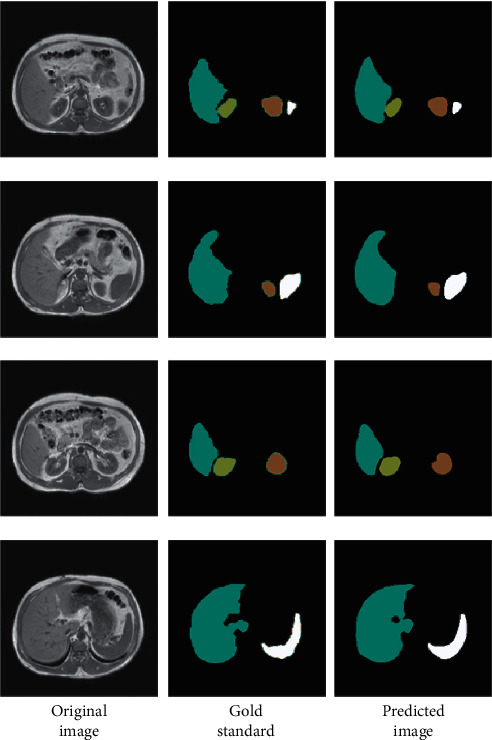
Comparison of model prediction segmentation and gold standard.

The AM-GAN training is carried out according to the algorithm strategy in Section 3 and the model structure and parameter setting in Section 4.2. Multitarget image segmentation is performed by generative network. Recall rate, precision, F1-score, accuracy, and Dice similarity coefficient are used to evaluate the properties of the segmentation model. The image segmentation experiment results of the test set are shown in Figure 11, and the index evaluation results are shown in Table 2.

In Figure 11, from left to right, there is the original abdominal image, the gold standard, that is, the image manually segmented by the expert, and the predicted segmented image by network. From Figure 11, the segmentation results of the GAN are clear and smooth. Whether it is a large-sized organ such as the liver or a small-sized organ such as the kidney and pancreas, the GAN can distinguish them more accurately, which shows that the proposed method has good applicability.

**Figure 12:**
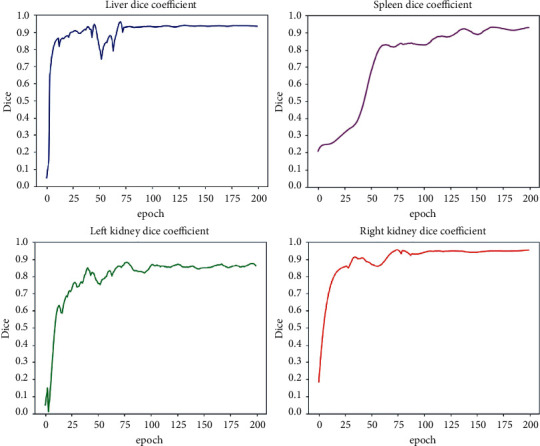
Variation curve of Dice coefficient of the segmented organ.

For analyzing the properties of the learning algorithm, the curves of the Dice coefficient indicators of four organs and tissues, including liver, left kidney, right kidney, and spleen changing with iteration, and the confusion matrix of the validation set are recorded. The results are shown in Figure 12 and Table 3.

In Figure 12, the Dice similarity coefficient training curves of the liver, left kidney, right kidney, and spleen are shown. The Dice curve of the liver converges quickly and is relatively stable in the later iterations. This is because its size is relatively large compared to other organs, which causes the segmentation network to be more inclined to learn the classification of the liver area pixels in the early stage. The left kidney, right kidney, and spleen organs are smaller in size than the liver, and the segmentation network has a deeper level, which leads to easy loss of information and large fluctuations in the training. In the later stage of training, the average Dice coefficient of liver, left kidney, right kidney, and spleen on the training set is 0.92, which shows that the overall effect of model segmentation is good, and it has good applicability to small-target segmentation.

From Table 3, the segmentation accuracy of liver organs is the highest, 97.55%. This is because the size of liver organs is relatively large, and the network tends to learn their pixel weights of liver organs. The spatial location of the left kidney and the liver area is relatively close, and the boundary tissues overlap, leading to the segmentation error of the left kidney mainly from the liver and background. The right kidney and spleen organs are close in space, leading to mutual influence. The right kidney and spleen are small-target organs, and there is no boundary overlap, so the segmentation is less affected than the left kidney. From the confusion matrix, the accuracy of left kidney segmentation was 82.39%, while that of right kidney segmentation was 92.68% and that of spleen segmentation was 94.12%. It shows that the method in this paper has a good ability to identify and distinguish the features of complex image pixel categories.

### 4.4. Comparative Experiment and Analysis

In order to verify the performance improvement of AM-GAN brought by the nonlocal attention mechanism, a ResNet-GAN model was constructed in the comparative experiment. This model removes the nonlocal attention mechanism in AM-GAN, and other structures are the same as the AM-GAN. In addition, current mainstream, AM-FCN [43] embedded based on attention, Attention U-Net [44] fused on attention in both encoder and decoder, DANet [45] embedded based on dual attention, and SEVNet [46] fused on SE module are selected as comparison models. The four index values of accuracy, recall, precision, and F1-score are used for comparative evaluation. In order to avoid misleading the performance evaluation by high background indicators, all evaluation indicators do not include the value of background indicators. The specific results are shown in Table 4.

From Table 4, the four indexes of AM-FCN and Attention U-Net are lower than the proposed model. This is because it organically integrates GAN's inherent ability to process and repair low-resolution images, and the global to local content association and feature enhancement mechanism of nonlocal attention improves the accuracy of multitarget segmentation results. Except for the recall rate, other indexes of DANet are lower than that of the proposed model, which shows that the nonlocal attention mechanism used in this model can gather the receptive field from global to local target area to realize information context correlation. In addition, GAN can optimize the segmentation results in mechanism and improve the segmentation properties of the model, and the model and algorithm are relatively robust. Compared with ResNet-GAN, the accuracy of proposed model is 1.1% higher, which shows that the nonlocal attention can effectively realize the information association from global to local. Combined with GAN's probability exploration mechanism, the segmentation accuracy of the model is comprehensively improved. Compared with the SEVNet, due to the inherent high-definition processing and repairing capabilities of the GAN for noise images and the use of a game strategy, the optimization of the segmentation results is realized, and the accuracy, continuity, and smoothness of the segmentation results are comprehensively improved. Based on the above analysis, it shows that the method in this paper has comprehensive advantages when performing image target segmentation with insignificant structural features and achieves a better segmentation effect.

## 5. Conclusion

In this paper, aiming at the fine segmentation of multitarget complex images, a generative adversarial network model fused with attention mechanism is proposed. In mechanism, the AM-GAN can use the ability to process and restore high-definition images of GAN to effectively reduce the effects of noise, offset distortion, and gray value distortion. Nonlocal spatial-channel dual attention is introduced to realize the information association and constraint of large receptive field content on local targets and maintain the continuity of segmentation results. At the same time, the Nash game strategy of the generative network and the adversarial network is adopted, which reduces the algorithm's loss of detailed features and effectively improves the accuracy of small-target segmentation. In terms of information processing mechanism, this method comprehensively realizes the context correlation of image information, the feature fusion of different levels and scales, the high-definition processing and repair of high-noise images, and the optimization of segmentation results. The experimental results show that the multitarget segmentation method proposed in this paper has good applicability for both small-size and large-size targets. Compared with the other methods, each evaluation index has been greatly improved. AM-GAN comprehensively utilizes the advantages of nonlocal attention mechanism and generative adversarial network, which can finely segment multi-instance targets in complex images. It has good applicability in mechanism to solve the image segmentation problem of insignificant morphological features and weak spatial information relevance. It improves the limitations and deficiencies of the comparison method in solving the above problems, provides a novel deep learning method for image segmentation, and has great application value and a good prospect for promotion. However, the information processing mechanism and algorithm process of the method in this paper are more complicated, and the image semantic knowledge and structural features are less used. From the experimental results, the proportion of image background pixels compared with target area pixels is too large, and the number of samples in the dataset is unbalanced, which still has a certain impact on the segmentation accuracy of this method. How to optimize the model and algorithm, improve the discrimination ability of the local target image and the background image, and embed the scene semantic feature knowledge into the segmentation model, which will be an important work in the next stage of research.

## Figures and Tables

**Algorithm 1:**
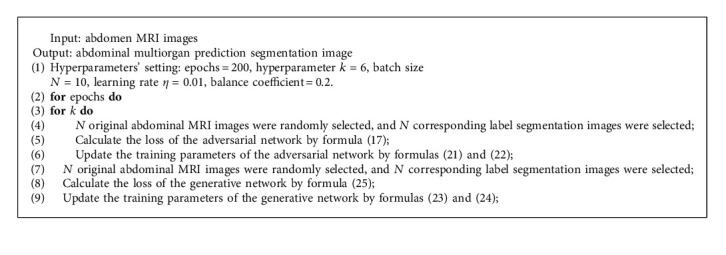
The AM-GAN training algorithm.

**Table 1:** Experimental dataset.

Slice type	Training set	Validation set	Test set	Total
Original slice	780	52	156	988
Extended slice	2340	52	156	2548
Total	3120	104	312	3536

**Table 2:** The evaluation results of AM-GAN.

Recall (%)	Precision (%)	F1-score (%)	Accuracy (%)
90.3	92.1	91.2	92.3

**Table 3:** Model numerical confusion matrix.

Type	Background	Liver	Left kidney	Right kidney	Spleen
Background	99.32%	0.59%	0.02%	0.02%	0.05%
Liver	1.89%	97.55%	0.24%	0.14%	0.18%
Left kidney	9.28%	7.73%	82.39%	0.29%	0.31%
Right kidney	4.72%	0%	0%	92.68%	2.6%
Spleen	4.71%	0%	0%	1.17%	94.12%

**Table 4:** Comparison of results of different models.

Model	Recall (%)	Precision (%)	F1-score (%)	Accuracy (%)
FCN	85.4	86.2	83.5	85.6
U-net	87.1	87.4	87.2	86.9
DANet	91.7	90.3	91.0	91.2
ResNet-GAN	89.2	91.5	90.1	91.2
SEVNet	86.5	87.6	86.8	87.1
Proposed	90.3	92.1	91.2	92.3

## Data Availability

The data that support the findings of this study are available upon request from the corresponding author.

## Supplementary Materials

Supplementary Materials The AM-GAN model combines the generative network based on the residual network and the nonlocal dual-attention mechanism with the adversarial network based on the CNNs to build a generative adversarial network model for the multitarget image segmentation. After AM-GAN is trained to reach the optimum, the model used for image segmentation is a generator network.
