# Supplementary material for: A Generative Adversarial Network Fused with Dual-Attention Mechanism and Its Application in Multitarget Image Fine Segmentation
Source: Comput Intell Neurosci. 2021 Dec 18;2021:2464648. doi: 10.1155/2021/2464648 (PMC8710171; doi:10.1155/2021/2464648)
Supplement: Supplementary Materials — The AM-GAN model combines the generative network based on the residual network and the nonlocal dual-attention mechanism with the adversarial network based on the CNNs to build a generative adversarial network model for the multitarget image segmentation. After AM-GAN is trained to reach the optimum, the model used for image segmentation is a generator network. [file 2464648.f1.docx]

The AM-GAN model. Combining the generative network based on the residual network and the non-local dual-attention mechanism with the adversarial network based on the CNNs to build a generative adversarial network model for the multi-target image segmentation. After AM-GAN is trained to reach the optimum, the model used for image segmentation is a generator network.
